# Further research on the clinical relevance of the ulcerative colitis colonoscopic index of severity for predicting 5-year relapse

**DOI:** 10.1007/s00384-021-04009-2

**Published:** 2021-08-18

**Authors:** Natsuki Ishida, Shunya Onoue, Takahiro Miyazu, Satoshi Tamura, Shinya Tani, Mihoko Yamade, Moriya Iwaizumi, Yasushi Hamaya, Satoshi Osawa, Takahisa Furuta, Ken Sugimoto

**Affiliations:** 1 First Department of Medicine, Hamamatsu University School of Medicine, 1-20-1 Handayama, Higashi-ku, Hamamatsu, Shizuoka 431-3192 Japan; 2 Department of Laboratory Medicine, Hamamatsu University School of Medicine, 1-20-1 Handayama, Higashi-ku, Hamamatsu, Shizuoka 431-3192 Japan; 3 Department of Endoscopic and Photodynamic Medicine, 1-20-1 Handayama, Higashi-ku, Hamamatsu, Shizuoka 431-3192 Japan; 4 Center for Clinical Research, Hamamatsu University School of Medicine, 1-20-1 Handayama, Higashi-ku, Hamamatsu, Shizuoka 431-3192 Japan

**Keywords:** Ulcerative colitis, Ulcerative colitis colonoscopic index of severity, Mayo endoscopic subscore, Clinical relapse

## Abstract

**Purpose:**

The ulcerative colitis colonoscopic index of severity (UCCIS) evaluates the state of the entire colonic mucosa in ulcerative colitis. However, no cut-off values of scores for predicting clinical relapse in patients with ulcerative colitis have been established. This study aimed to determine the cut-off values for predicting clinical relapse in patients with ulcerative colitis.

**Methods:**

The endoscopic scores (sum of Mayo endoscopic subscores (S-MES) and UCCIS) of 157 patients with ulcerative colitis experiencing clinical remission and their subsequent clinical course were retrospectively reviewed. The optimal cut-off values for predicting relapse and relapse-free rates were analyzed by receiver operating characteristic analysis.

**Results:**

Forty patients with ulcerative colitis experienced relapse within 24 months. The median UCCIS for these patients at the time of study enrollment was significantly higher than that for patients with clinical remission (*P* < 0.001). The cut-off value of the UCCIS for predicting relapse was 9.8. The relapse-free rate was significantly lower in patients with UCCIS ≥ 9.8 than in those with UCCIS < 9.8 (log-rank test *P* < 0.001). For patients who experienced relapse within 5 years, the optimal cut-off values for the UCCIS and S-MES were 10.2 and 1, respectively (*P* = 0.004).

**Conclusions:**

The data from this study indicate that the USSIC is a more relevant score than the S-MES for predicting the time to relapse in patients with ulcerative colitis in remission.

**Supplementary information:**

The online version contains supplementary material available at 10.1007/s00384-021-04009-2.

## Introduction

Ulcerative colitis (UC) is an inflammatory bowel disease characterized by repeated relapses and remissions [1]. The evaluation of UC using an endoscopic score is important for establishing the patient’s condition. The Mayo endoscopic subscore (MES) is one of the most commonly used endoscopic scores in clinical practice and trials [2]. Mucosal healing is one goal of treatment and enables long-term clinical remission and the reduction of hospitalization rates and risk of surgical treatment [3]. Patients with UC with MES 1 reportedly have a higher risk of relapse than do those with MES 0 [4–6]. Biomarker assessment for UC is an alternative to colonoscopy, and some biomarkers, such as fecal calprotectin and fecal immunochemical occult blood tests, can predict clinical relapse in patients with UC [7–13]. In addition, an observational study using fecal calprotectin showed that treatment intensification based on fecal calprotectin levels improved outcomes in patients with UC in clinical remission; this suggests that relapse prediction is important in the treatment of UC [14, 15].

The MES is a score that identifies the area with the most severe colitis in patients with UC. The ulcerative colitis endoscopic index of severity (UCEIS), often used in clinical practice and trials, also reveals the area with the most severe colitis [16]. The UCEIS is calculated by adding the scores of descriptors of vascular pattern, bleeding, and erosions and ulcers, although the calculation process is more complex than that of the MES. Other endoscopic scores evaluating the state of the entire colon have been reported [17, 18]. One such score, the S-MES, is calculated by assessing the MES of five segments of the colon (ascending colon, transverse colon, descending colon, sigmoid colon, and rectum) and summing the scores [17]. In a comparative study, the S-MES correlated more strongly with fecal calprotectin than did the conventional MES [17]. Other studies have reported correlations between the ulcerative colitis colonoscopic index of severity (UCCIS) and the clinical activity index (CAI) and other findings, including the C-reactive protein concentration [19–21]. The UCCIS is an endoscopic score that reveals the inflammation of the entire colon and is similar to the UCEIS. The UCCIS is calculated from the scores of four descriptors of granularity, in addition to the vascular pattern, bleeding, and erosions and ulcers which are descriptors used to calculate the UCEIS.

Although the UCCIS scoring process is more complex than conventional MES and S-MES calculations, it may more precisely reflect the inflammatory state of the intestinal mucosa. However, the cut-off value for clinical relapse based on the UCCIS has not been established; hence, the UCCIS is rarely used in clinical practice.

This study aimed to determine the UCCIS and S-MES cut-off values that predict clinical relapse and examine the clinical usefulness of these scores.

## Methods

### Patients and study design

Patients with UC treated at the Hamamatsu University School of Medicine between April 2012 and November 2020 were enrolled in this single-center, retrospective cohort study. These patients met the following criteria for at least 3 months: clinical remission (CAI [Rachmilewitz index] ≤ 3) and mucosal healing (MES ≤ 1). UC was diagnosed according to current guidelines, with typical clinical symptoms and endoscopic and histological evaluations [22]. Patients with no diagnosis of UC, such as those with an indeterminate colitis diagnosis or unidentified inflammatory bowel disease, were excluded. Because it was essential to evaluate the entire colon via colonoscopy in this study, patients in whom it was not possible to visualize the colon from the anus to the cecum and patients who had undergone colectomy were excluded.

In this study, the primary endpoint was the UCCIS cut-off value that predicts UC relapse. The secondary endpoint was a comparison of the accuracy of the UCCIS and S-MES for predicting UC relapse.

### Disease assessment

We evaluated clinical disease activity using the CAI, according to Rachmilewitz [23]. Herein, clinical remission was defined as a CAI of ≤ 3.

### Endoscopic assessment

The enrolled patients with UC underwent bowel preparation through the application of a polyethylene glycol-based electrolyte solution before colonoscopic examination. All images of the areas in each segment where inflammation was most prominent were evaluated by endoscopic scoring. The state of the colonic mucosa was evaluated using the MES, S-MES, UCEIS, and UCCIS.

The MES was assessed using the following conventional criteria: 0, normal or inactive disease; 1, mild disease with erythema, decreased vascular pattern, and mild friability; 2, moderate disease with marked erythema, absence of vascular patterns, friability, and erosions; and 3, severe disease with spontaneous bleeding and ulceration. Mucosal healing was defined as MES 0 or 1 [2]. The MES identifies the colonic lesions with the most severe inflammation, and the highest MES score was defined as the maximum MES (M-MES). The S-MES was calculated by determining the MES of each of the five segments (ascending colon, transverse colon, descending colon, sigmoid colon, and rectum) according to the above criteria, and the scores were then added.

The UCEIS score was calculated by adding the following descriptors: vascular pattern (score 0–2), bleeding (score 0–3), and erosions and ulcers (score 0–3) [14]. Similar to the M-MES, these descriptors were evaluated at the most active lesions of the colon. The UCEIS score ranged from 0 to 8 points. The UCCIS scores were first assessed in the five segments using the following descriptors: vascular pattern (score 0–2), granularity (score 0–2), erosions and ulcers (score 0–4), and bleeding/friability (score 0–2). The scores of these descriptors were entered into the following formula [19, 20]: UCCIS = (3.1 × the sum (vascular pattern across the five segments)) + (3.6 × the sum (granularity across the five segments)) + (3.5 × the sum (ulceration across the five segments)) + (2.5 × the sum (bleeding/friability across the five segments)).

These four endoscopic scores were evaluated by five expert gastroenterologists (NI, TM, S Tamura, S Tani, and KS). These physicians were blinded to the patients’ clinical information, including the prognosis. To determine the endoscopic score in each segment, each expert reviewed and scored all images. If there were differences in the scores among the experts, this was resolved by consensus.

### Patient follow-up

The enrolled patients visited our facility every 1–3 months. They were instructed to record their clinical symptoms, based on the CAI, to monitor their daily condition. Clinical relapse was defined as the need to modify, change, or supplement UC treatment due to increased bowel movement, bloody stools, and a CAI of ≥ 4 for the first time after endoscopic examination. Treatment was initiated at the discretion of the attending physician.

### Statistical analysis

Statistical analyses of the data were performed using IBM SPSS Statistics for Windows, version 24 (IBM Corp., Armonk, NY, USA) and EZR (Saitama Medical Center, Jichi Medical University, Saitama, Japan) software. The Mann–Whitney *U* test was used to evaluate differences. The optimal S-MES and UCCIS cut-off values for predicting clinical relapse were analyzed using receiver operating characteristic (ROC) analyses. The accuracy of the predictive values was determined using the area under the ROC curve (AUC). Kaplan–Meier analysis with the log-rank test and Cox hazard ratio multivariate analysis were used to evaluate the cumulative rate of relapse-free survival. *P* < 0.05 were considered significant.

### Ethical considerations

This retrospective study protocol was reviewed and approved by the Ethics Committee of Hamamatsu University School of Medicine (reference number 20–322), and the study was conducted according to Good Clinical Practice Principles in compliance with the Declaration of Helsinki. The requirement for informed consent was waived owing to the retrospective nature of the study and the utilization of anonymous data.

## Results

### Patient characteristics

**Fig. 1:**
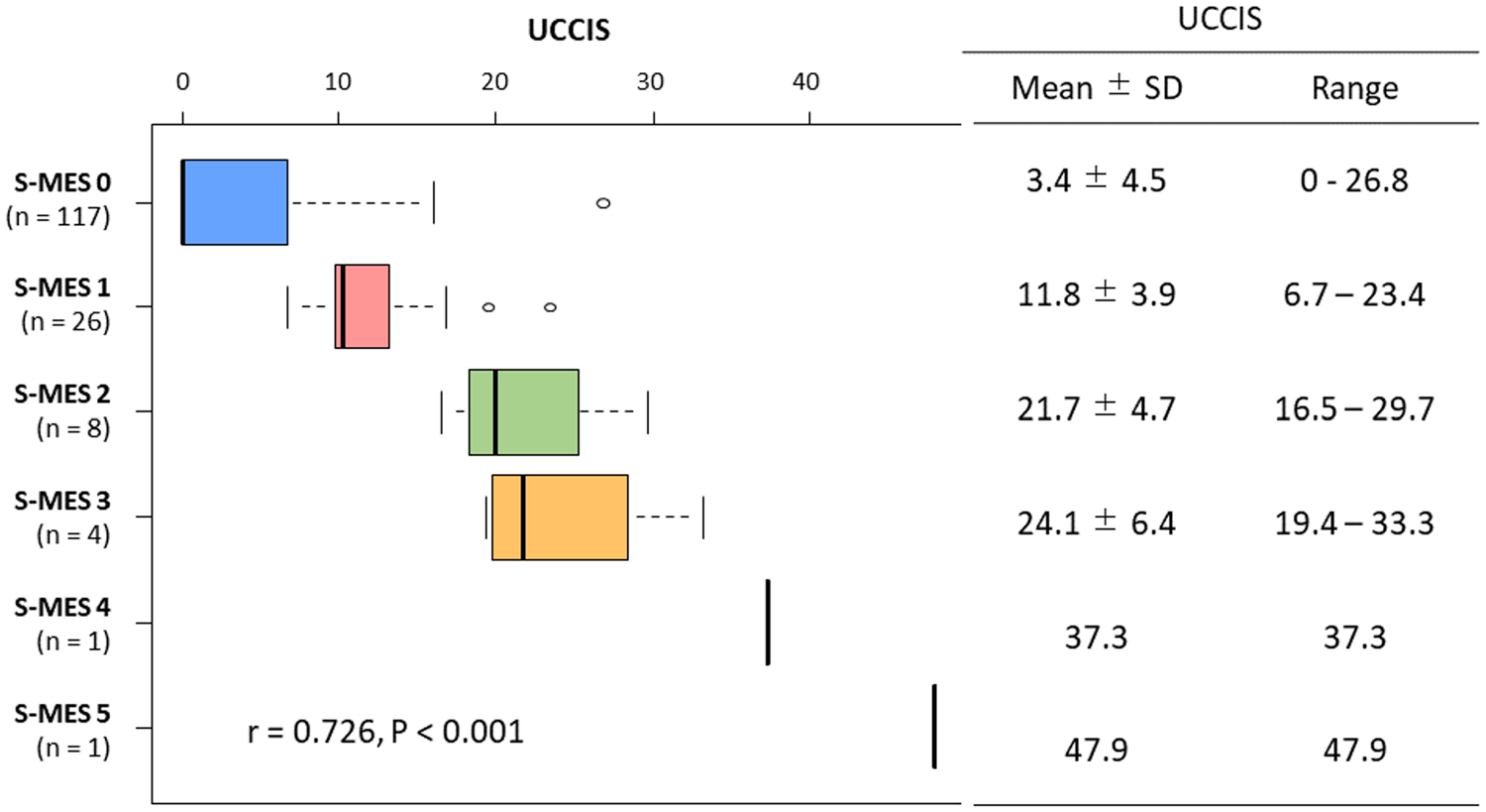
Correlation between the sum of the Mayo endoscopic subscores (S-MES) and the ulcerative colitis colonoscopic index of severity (UCCIS). A significant correlation was found between the two endoscopic scores (*r* = 0.726, *P* < 0.001). The right table shows the mean ± standard deviation (SD) and the range of the UCCIS in each S-MES group

A total of 157 patients (mean [range] age 47.2 [18–84] years; mean [range] disease duration: 9.6 [0.3–38] years) with UC who underwent total colonoscopy were enrolled in this study (Table 1). The mean scores of the M-MES, S-MES, UCEIS, and UCCIS were 0.25, 0.40, 0.70, and 6.71, respectively. A significant correlation was found between the S-MES and the UCCIS (*r* = 0.726, *P* < 0.001) (Fig. 1). The median observation period in this study was 887 days.

**Table 1:** Characteristics of patients with ulcerative colitis in clinical remission

Characteristics		*N* = 157	
Age (years)		47.2 (18–84) ± 16.8	
Male/female, *n* (%)		99/58 (63.1/36.9)	
Disease duration (years)		9.6 (0.3–38) ± 8.6	
Disease extent, *n* (%)	Extensive colitis Left-sided colitis Proctitis		100 (63.7) 40 (25.5) 17 (10.8)
CAI (Rachmilewitz index)		0.47 (0–3) ± 0.92	
M-MES		0.25 (0–1) ± 0.44	
S-MES		0.40 (0–5) ± 0.84	
UCEIS		0.70 (0–5) ± 0.91	
UCCIS		6.71 (0–81.2) ± 8.25	
Medication at study, *n* (%)	Oral 5-ASA Suppository 5-ASA Systemic steroids Immunomodulators Biologics		119 (75.8) 16 (10.2) 21 (13.4) 49 (31.2) 22 (14.0)

Data are presented as the mean (range) ± standard deviation unless otherwise noted

*5-ASA* 5-aminosalicylic acid, *CAI* clinical activity index, *M-MES* maximum Mayo endoscopic subscore, *S-MES* sum of Mayo endoscopic subscores, *UCEIS* ulcerative colitis endoscopic index of severity, *UCCIS* ulcerative colitis colonoscopic index of severity

### Comparison of patients with relapse and clinical remission within 2 years of colonoscopy

**Fig. 2:**
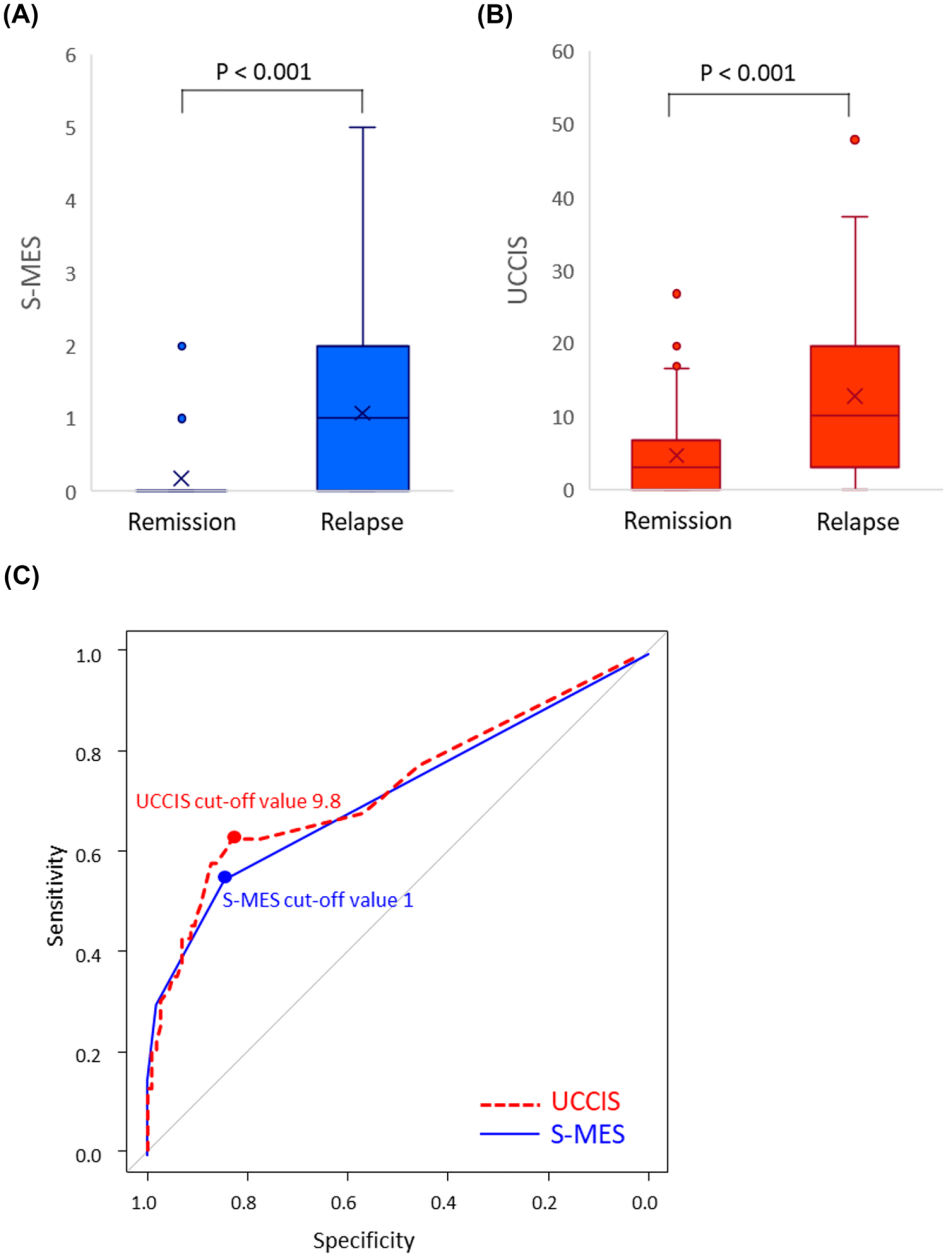
Sum of the Mayo endoscopic subscores (S-MES) and ulcerative colitis colonoscopic index of severity (UCCIS) scores during a 2-year follow-up period and their utility in predicting future clinical relapse in patients with ulcerative colitis (UC). (**A)** Difference in the S-MES between patients with UC at relapse and those with clinical remission during a 2-year follow-up period. (**B)** Difference in the UCCIS score between patients with UC at relapse and those with clinical remission during a 2-year follow-up period. (**C)** Receiver-operating characteristic analysis using the S-MES and UCCIS for the prediction of future clinical relapse in patients with UC who were experiencing clinical remission at the time of enrollment

Forty patients (25.4%) with UC experienced clinical relapse during the 2-year post-colonoscopy follow-up. The median S-MES and UCCIS scores of patients who experienced relapse were significantly higher than those of patients who experienced clinical remission (both *P* < 0.001) (Fig. 2A, B). The ROC analyses of the clinical course of patients who experienced relapse in the 2-year follow-up period and those of patients who experienced remission showed that the S-MES and UCCIS cut-off scores for predicting clinical relapse were 1 and 9.8, respectively, and the AUCs were 0.718 (95% confidence interval [CI] 0.630–0.806) and 0.727 (95% CI 0.626–0.828), respectively. No significant difference was found between the AUCs of the S-MES and UCCIS (*P* = 0.763) (Fig. 2C). In this ROC analysis, the sensitivity and specificity of the S-MES for 2-year relapse prediction were 55.0% and 84.6%, respectively, and those of the UCCIS were 62.5% and 82.9%, respectively.

### Comparison of the S-MES and UCCIS for the prediction of subsequent relapse within 2 years of colonoscopy

**Fig. 3:**
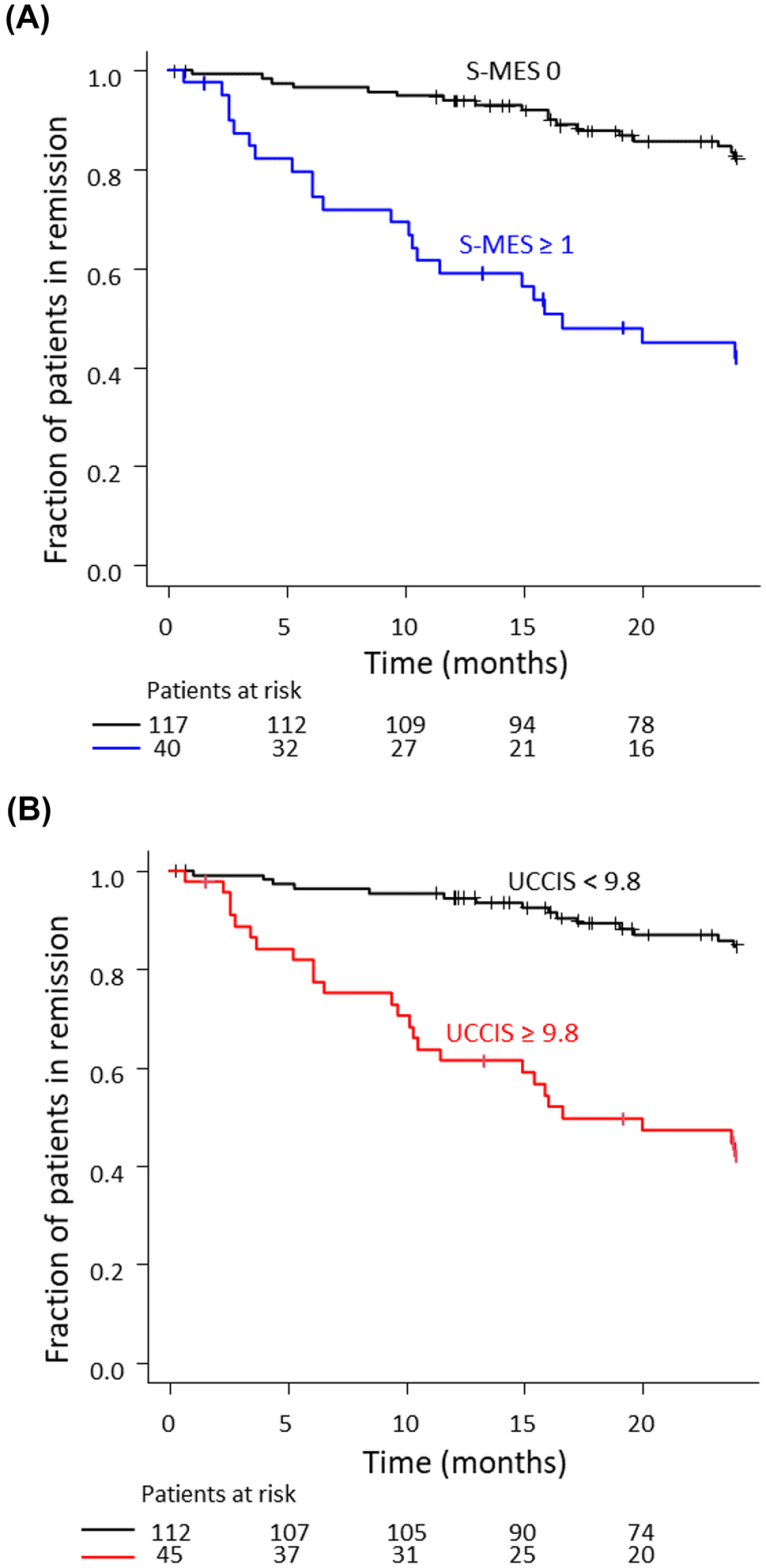
Kaplan–Meier time-to-relapse curves during a 2-year follow-up period. Kaplan–Meier time-to-relapse curve of patients with ulcerative colitis (UC) during a 2-year follow-up period in relation to the (**A)** sum of Mayo endoscopic subscores (S-MES) (S-MES 0 versus S-MES ≥ 1) and (**B)** ulcerative colitis colonoscopic index of severity (UCCIS) (UCCIS < 9.8 versus UCCIS ≥ 9.8). These Kaplan–Meier curves show significant differences in each group (*P* < 0.001 and *P* < 0.001, respectively)

Remission-free survival was compared between the group of patients with S-MES 0 and S-MES ≥ 1 using Kaplan–Meier analysis (Fig. 3A). There were 117 and 40 patients with S-MES 0 and S-MES ≥ 1, respectively, and 18 of the 117 patients (15.4%) and 22 of the 40 patients (55.0%) experienced relapse. The relapse rate in the S-MES ≥ 1 group was significantly higher than that in the S-MES 0 group (log-rank test *P* < 0.001) at 2 years after colonoscopy. We performed Kaplan–Meier analysis of remission-free survival in the UCCIS ≥ 9.8 and UCCIS < 9.8 groups (Fig. 3B). There were 112 and 45 patients with UC in the UCCIS ≥ 9.8 and UCCIS < 9.8 groups, respectively, and 15 of the 112 patients (13.4%) and 25 of the 45 patients (55.6%) experienced clinical relapse within 2 years. The relapse rate in the UCCIS ≥ 9.8 group was significantly higher than that in the UCCIS < 9.8 group at 2 years after colonoscopy (log-rank test *P* < 0.001).

### Comparison of patients with relapse and clinical remission within 5 years of colonoscopy

**Fig. 4:**
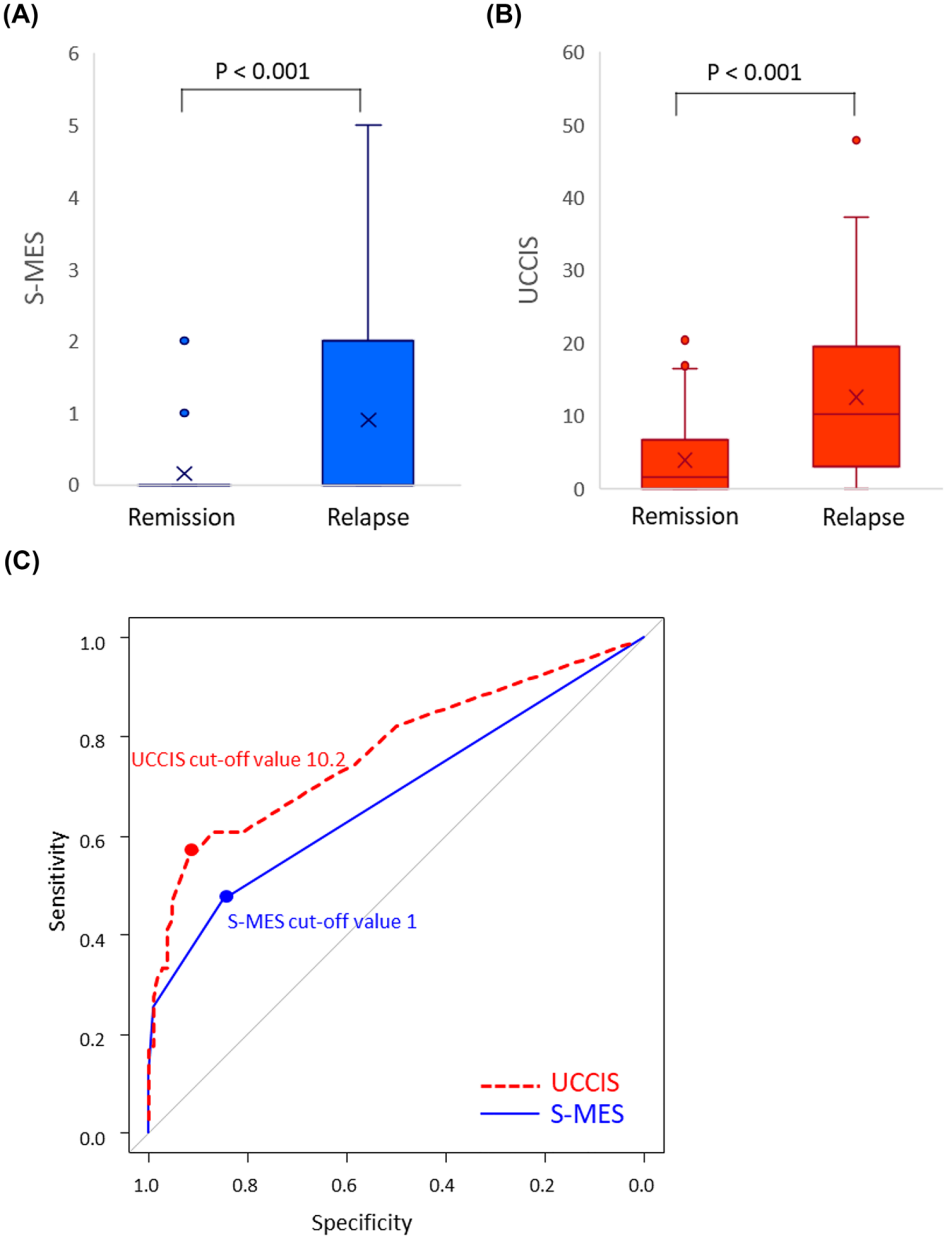
Sum of the Mayo endoscopic subscores (S-MES) and ulcerative colitis colonoscopic index of severity (UCCIS) scores during a 5-year follow-up period and their utility in predicting future clinical relapse in patients with ulcerative colitis (UC). (**A)** Difference in the S-MES between patients with UC at relapse and those with clinical remission during a 5-year follow-up period. (**B)** Difference in the UCCIS score between patients with UC at relapse and those experiencing clinical remission during a 5-year follow-up period. (**C)** Receiver-operating characteristic analysis using the S-MES and UCCIS for the prediction of future clinical relapse in patients with UC experiencing clinical remission at the time of enrollment

We extended the observation period from 2 to 5 years and repeated the abovementioned analyses (Fig. 4). There were 51 cases of clinical relapse within the 5-year period. The median S-MES and UCCIS scores for patients who experienced relapse were significantly higher than those of patients who experienced clinical remission during the 5-year period (both *P* < 0.001) (Fig. 4A, B). The S-MES and UCCIS cut-off scores for predicting clinical relapse within 5 years of colonoscopy were 1 and 10.2, respectively. Notably, the AUC of the UCCIS (0.772 [95% CI: 0.689–0.856]) was significantly higher than that of the S-MES (0.677 [95% CI 0.598–0.757]) (*P* = 0.004) (Fig. 4C). The sensitivity and specificity of the S-MES prediction of 5-year relapse were 51.1% and 84.8%, respectively, and those of the UCCIS were 56.9% and 91.5%, respectively.

### Comparison of patients with UCCIS ≥ 10.2 and UCCIS < 10.2 within 5 years of colonoscopy

**Fig. 5:**
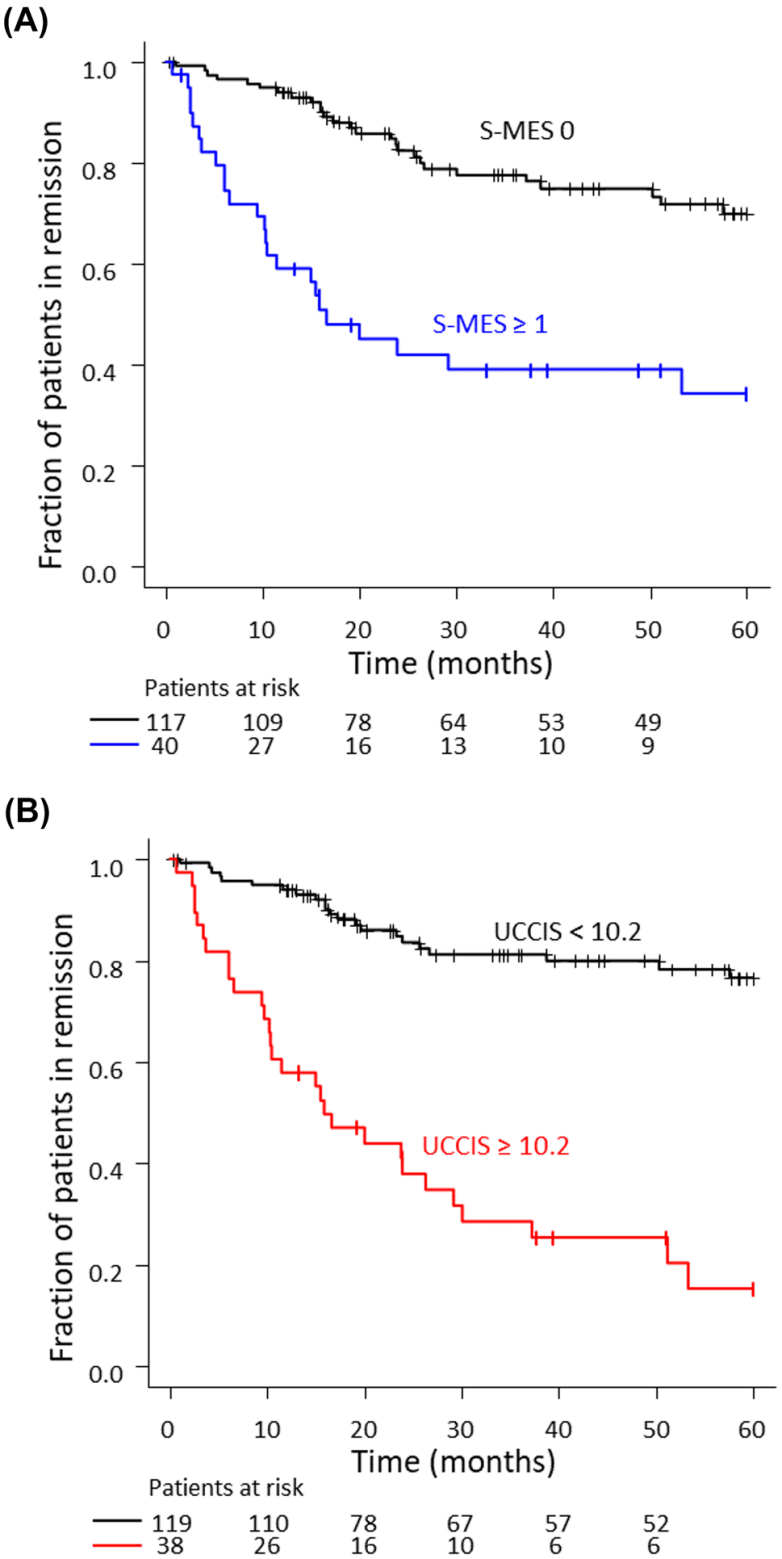
Kaplan–Meier time-to-relapse curves during a 5-year follow-up period. Kaplan–Meier time-to-relapse curve of patients with ulcerative colitis (UC) during a 5-year follow-up period in relation to the (**A)** sum of the Mayo endoscopic subscores (S-MES) (S-MES 0 versus S-MES ≥ 1) and (**B)** ulcerative colitis colonoscopic index of severity (UCCIS) (UCCIS < 10.2 versus UCCIS ≥ 10.2). These Kaplan–Meier curves show significant differences in each group (*P* < 0.001 and *P* < 0.001, respectively)

Finally, we analyzed the remission-free survival in the S-MES and UCCIS groups using the Kaplan–Meier curve over a 5-year observation period (Fig. 5). The relapse rate in the S-MES ≥ 1 group (60.0%) was significantly higher than that in the S-MES 0 group (23.1%) (log-rank test *P* < 0.001) (Fig. 5A). The relapse rate in the UCCIS ≥ 10.2 group (76.3%) was significantly higher than that in the UCCIS < 10.2 group (18.5%), and the difference in the UCCIS relapse rate was more pronounced than the difference in the S-MES relapse rate (log-rank test *P* < 0.001) (Fig. 5B). Multivariate analysis was additionally performed using the Cox proportional hazards model (Table 2). Analysis including other factors showed that a UCCIS of ≥ 10.2 was an independent factor related to relapse. Other independent factors associated with relapse were female sex, age, non-use of immunomodulatory agents, and use of biologics.

**Table 2:** Multivariate analysis for predicting clinical relapse during a 5-year follow-up period in patients with ulcerative colitis experiencing remission

		Univariate analysis			Multivariate analysis		
Variable		Hazard ratio	95% CI	*P*-value	Hazard ratio	95% CI	*P*-value
UCCIS ≥ 10.2		6.391	3.645–11.21	< 0.001	6.142	3.348–11.27	< 0.001
Male sex		0.473	0.273–0.820	0.008	0.484	0.274–0.856	0.013
Age		0.972	0.954–0.991	0.003	0.974	0.954–0.993	0.009
Disease extent	Extensive colitis Left-sided colitis Proctitis	1.320 1.062 0.318	0.731–2.386 0.574–1.964 0.543–1.982	0.357 0.849 0.112	3.008 4.191 NA	0.693–13.06 0.914–19.21 NA	0.142 0.065 NA
Medication at study	Oral 5-ASA Suppository 5-ASA Systemic steroids Immunomodulators Biologics	1.037 1.013 3.243 0.630 2.153	0.077–1.308 0.4024–2.550 1.718–6.123 0.330–1.204 1.101–4.210	0.911 0.978 <0.001 0.162 0.025	1.112 0.593 1.954 0.461 3.024	0.534–2.317 0.534–2.317 0.947–4.030 0.223–0.952 1.374–6.655	0.777 0.303 0.070 0.036 0.006

*5-ASA* 5-aminosalicylic acid, *CI* confidence interval, *NA* not available, *UCCIS* ulcerative colitis colonoscopic index of severity

## Discussion

This study investigated whether clinical relapse can be predicted by the UCCIS, an endoscopic score that reflects the inflammation of the entire colon. Significant differences were found in the UCCIS scores between the remission and relapse groups, and these scores were markers for predicting UC relapse over the 2-year follow-up period. The UCCIS was compared with the S-MES, which is a simple sum of the MES of five colon segments. Herein, S-MES 0 versus S-MES ≥ 1 was equivalent to M-MES 0 versus M-MES 1, as we only targeted patients with M-MES 0 and patients with M-MES 1 (i.e., conventional MES 0 and MES 1, respectively). This suggests that if S-MES is 0, then M-MES is also 0. In addition, as the S-MES cut-off score was exactly 1 in this study, verifying the prediction of relapse using the S-MES is equivalent to the conventional comparison of MES 0 versus MES 1, which is a criterion for determining mucosal healing. Moreover, consistent with previous reports that MES 1 is associated with relapse [4–6], our results indicate that patients with S-MES ≥ 1 are at risk of relapse. In our study, the AUC of the UCCIS tended to be higher than that of the S-MES, and the high accuracy of the UCCIS was confirmed. Analysis using 9.8 as the cut-off value of the UCCIS showed that remission-free survival was significantly lower in the UCCIS ≥ 9.8 group than in the UCCIS < 9.8 group, demonstrating that the UCCIS is also useful as a predictor of relapse.

The evaluation method of the UCCIS is complicated, and it was necessary to determine its advantage over the simple MES evaluation methods. Our 2-year ROC analysis to determine the usefulness of the UCCIS in predicting the short- to medium-term prognosis did not show a significant difference in the AUC between the UCCIS and the S-MES. When the observation period was extended to 5 years, the UCCIS cut-off value for relapse prediction was 10.2, and the AUC of the UCCIS was significantly greater than that of the S-MES. The specificity was high for the 2-year and 5-year follow-ups, and the sensitivity and specificity of the UCCIS were high in the 5-year relapse prediction. This significant difference in AUC is attributed to the ability of the UCCIS to provide more detailed information about the inflammatory condition of the colon, relative to the information provided by the S-MES. Regarding the S-MES, an MES of 0 reflects minimal inflammation, while an MES of 1 reflects mild to relatively severe inflammation. Compared with the MES, the UCCIS can detect more detailed differences in the inflammatory by evaluating the vascular pattern, granularity, erosions and ulcers, and bleeding/friability. Furthermore, by evaluating these descriptors in the entire colon, the difference becomes even more pronounced. As shown in Fig. 1, the range of UCCIS corresponding to S-MES 0, S-MES 1, S-MES 2, and S-MES 3 is 0–26.8, 6.7–23.4, 16.5–29.7, and 19.4–33.3, respectively. Accordingly, the range from the maximum to the minimum UCCIS value is wide, even within the same S-MES group. It was suggested that the UCCIS was more useful than the S-MES for predicting long-term relapse (5 years) because it could detect inflammatory states of the intestinal tract in detail. The UCEIS, which like the UCCIS is evaluated by descriptors, is reportedly useful for predicting the medium- to long-term prognosis of UC [24, 25].

It is recommended that endoscopic scores have a cut-off value that reflects the clinical condition of the patient. The most important (or relevant) parameter was the prediction of clinical relapse using MES 0 versus MES 1 [4–6]. However, the UCCIS cut-off value for predicting clinical relapse has not been defined. Although endoscopic scores evaluating inflammatory changes in the entire colon have been reported, most of the inflammatory changes were compared with clinical symptoms, blood test findings, and biomarkers [17–20, 26, 27], and most of the endoscopic scores lacked cut-off values for ascertaining the extent of UC and for determining the prognosis.

The UCCIS evaluation method was reported in 2012, but it is not widely used in clinical practice and trials due to the complexity of the scoring system [19]. To evaluate the four descriptors in each of the five segments of the intestine, 20 items need to be scored, and the calculation is based on substitution. Therefore, calculating the UCCIS score requires time and effort. Recently, endoscopic evaluation using artificial intelligence (AI) has been reported in gastrointestinal endoscopy, and AI has been used for diagnosis and disease assessment in UC [28–30]. Although evaluation of the entire colon using an endoscopic score, such as the UCCIS, tends to be avoided due to the complexity of the calculation method in clinical practice, if the state of the entire colonic mucosa could be automatically evaluated by AI, an endoscopic score evaluating the entire colon could be used more commonly in clinical practice.

This study has some limitations. First, this retrospective study was performed at a single center. Second, endoscopic evaluation was performed by using images taken in the past. Third, we did not collect data regarding relapse of patients prior to their enrollment in this study, and this may have influenced the study results. Fourth, the UCCIS and S-MES were not compared with biomarkers or histological evaluations. Many studies have compared relapse prediction with biomarkers and histological scores, and the combination of fecal calprotectin and fecal immunochemical tests has enabled more accurate relapse prediction [7–13]. Histological evaluations of UC also predict relapse, and mucin depletion is a risk factor for relapse [31]. Future prospective studies should evaluate the UCCIS in combination with biomarkers and histological scores for a more accurate prediction of prognosis.

In conclusion, the UCCIS is a reliable endoscopic score for predicting clinical relapse in patients with UC and is more useful than the MES for predicting mid- to long-term clinical relapse.

## Supplementary information

Below is the link to the electronic supplementary material.

Supplementary file1 (DOC 38 KB)

## Data Availability

Not applicable.
