# Supplementary material for: Further research on the clinical relevance of the ulcerative colitis colonoscopic index of severity for predicting 5-year relapse
Source: Int J Colorectal Dis. 2021 Aug 18;36(12):2661–70. doi: 10.1007/s00384-021-04009-2 (PMC8589779; doi:10.1007/s00384-021-04009-2)
Supplement: Supplementary file 1 — Supplementary file1 (DOC 38 KB) [file 384_2021_4009_MOESM1_ESM.doc]

June 12, 2021

Christoph-Thomas Germer

Editor-in-Chief

*International Journal of Colorectal Disease*

Dear Editor:

We wish to submit an Original Article for publication in *International Journal of Colorectal Disease*, titled “**Ulcerative colitis colonoscopic index of severity: a useful predictor of 5-year clinical relapse**.” The paper was coauthored by Natsuki Ishida, Shunya Onoue, Takahiro Miyazu, Satoshi Tamura, Shinya Tani, Mihoko Yamade, Moriya Iwaizumi, Yasushi Hamaya, Satoshi Osawa, and Takahisa Furuta.

The ulcerative colitis colonoscopic index of severity (UCCIS) is an endoscopic score that reflects inflammation of the entire colon of patients with ulcerative colitis (UC), although its cut-off value for predicting clinical relapse has not been determined. In this single-center, retrospective cohort study, we established the cut-off value of UCCIS for predicting clinical relapse in patients with UC in clinical remission and compared this with the value for the sum of Mayo endoscopic subscores (S-MES).

We believe that our study makes a significant contribution to the literature because it shows that the UCCIS is a reliable endoscopic score that can predict clinical relapse at cut-off values of 9.8 and 10.2 for 2-year and 5-year follow-up periods, respectively. Further, we demonstrate that the accuracy of relapse prediction using the UCCIS was higher than that using the S-MES at the 5-year follow-up.

We believe that this article will be suitable for the general readership of your journal because it contains clinical research in the field of gastroenterology and identifies, for the first time, that a UCCIS cut-off value of 10.2 or higher is an independent relapse factor, which has important implications for clinical practice.

Please consider, as potential referees: Dr. Abbi Saniabadi (JIMRO, abbirs@hotmail.co.uk), Dr. Kentaro Ikeya (Tanaka gastrointestinal clinic, ki.key.adr@gmail.com), Dr. Shunji Ishihara (Shimane University, si360405@med.shimane-u.ac.jp).

All authors made substantial contributions to the manuscript, including final approval of the version to be submitted. This manuscript, including related data, figures, and tables, has not been published or presented elsewhere in part or in entirety and is not under consideration by any other journal. The study design was approved by the appropriate ethics review board and the requirement for informed consent was waived due to the retrospective study design. We have read and understood your journal’s policies, and we believe that neither the manuscript nor the study violates any of these. There are no conflicts of interest to declare.

Thank you for your consideration. I look forward to hearing from you.

Sincerely,

Ken Sugimoto, MD, PhD

The First Department of Medicine

Hamamatsu University School of Medicine

1-20-1 Handayama, Higashi-ku, Hamamatsu 431-3192, Shizuoka, Japan

Tel: +81-53-435-2261

Fax: +81-53-434-9447

Email: sugimken@hama-med.ac.jp
